# Looking downstream: the role of cyclic AMP-regulated genes in axonal regeneration

**DOI:** 10.3389/fnmol.2015.00026

**Published:** 2015-06-18

**Authors:** Mustafa M. Siddiq, Sari S. Hannila

**Affiliations:** ^1^Icahn Medical Institute, Pharmacology and Systems Therapeutics, Mount Sinai School of MedicineNew York, NY, USA; ^2^Department of Human Anatomy and Cell Science, University of ManitobaWinnipeg, MB, Canada

**Keywords:** cyclic AMP, transcription, arginase I, interleukin-6, SLPI, metallothionein, regeneration, spinal cord injury

## Abstract

Elevation of intracellular cyclic AMP (cAMP) levels has proven to be one of the most effective means of overcoming inhibition of axonal regeneration by myelin-associated inhibitors such as myelin-associated glycoprotein (MAG), Nogo, and oligodendrocyte myelin glycoprotein. Pharmacological manipulation of cAMP through the administration of dibutyryl cAMP or rolipram leads to enhanced axonal growth both *in vivo* and *in vitro*, and importantly, upregulation of cAMP within dorsal root ganglion neurons is responsible for the conditioning lesion effect, which indicates that cAMP plays a significant role in the endogenous mechanisms that promote axonal regeneration. The effects of cAMP are transcription-dependent and are mediated through the activation of protein kinase A (PKA) and the transcription factor cyclic AMP response element binding protein (CREB). This leads to the induction of a variety of genes, several of which have been shown to overcome myelin-mediated inhibition in their own right. In this review, we will highlight the pro-regenerative effects of arginase I (ArgI), interleukin (IL)-6, secretory leukocyte protease inhibitor (SLPI), and metallothionein (MT)-I/II, and discuss their potential for therapeutic use in spinal cord injury.

## Cyclic AMP: Overcoming Inhibition by Inducing Transcription

The identification of myelin-associated glycoprotein (MAG), Nogo, and oligodendrocyte myelin glycoprotein as inhibitors of neurite outgrowth was a turning point in the study of axonal regeneration, providing compelling evidence that CNS myelin proteins contributed to regenerative failure after injury. Not surprisingly, their discovery sparked great interest in developing experimental approaches to overcome this inhibition, and one approach in particular has provided unique insight into the intrinsic mechanisms that regulate axonal regeneration. Soon after the conditioning lesion effect was described by Neumann and Woolf (1999), the laboratory of Dr. Marie T. Filbin demonstrated that elevation of intracellular cyclic AMP (cAMP) levels through the administration of dibutyryl cAMP (dbcAMP) reverses inhibition of neurite outgrowth by MAG and CNS myelin (Cai et al., 1999). In subsequent studies it was shown that elevation of cAMP underlies the conditioning lesion effect, as cAMP levels became elevated in dorsal root ganglia (DRG) in response to a sciatic nerve lesion, and injection of dbcAMP directly into DRG replicated the effects of a conditioning lesion on dorsal column axon regeneration (Neumann et al., 2002; Qiu et al., 2002). The most important implication of these findings was that cAMP activates an intrinsic pro-regenerative program within the neuron that allows it to overcome MAG- and myelin-mediated inhibition at the molecular level.

Experiments aimed at characterizing this mechanism soon revealed that the effects of cAMP were dependent on the activation of protein kinase A (PKA), as administration of the PKA inhibitor KT5720 abolished the ability of dbcAMP to overcome inhibition by MAG (Qiu et al., 2002). One of the primary downstream targets of PKA is the transcription factor cyclic AMP response element binding protein (CREB), which is phosphorylated at Ser133 and recruits the co-activator CREB-binding protein (CBP)/p300, which in turn leads to initiation of transcription by RNA polymerase II (Mayr and Montminy, 2001). Interestingly, there is evidence that like CREB, CBP/p300 activity can be regulated by PKA-mediated phosphorylation (Vo and Goodman, 2001), and other kinases such as cyclin E/Cdk2, calcium/calmodulin kinase IV, and mitogen-activated protein kinase have also been shown to play a role in the regulation of CBP/p300 (Vo and Goodman, 2001). CBP also facilitates transcription through its histone acetyltransferase activity, and it has been shown that histone hyperacetylation mediated through inhibition of histone deacetylases enhances neurite outgrowth for cerebellar neurons plated on CNS myelin substrates (Gaub et al., 2010). This response is dependent on CPB/p300 activity, as siRNA knockdown of this protein abolished the ability of neurons to overcome inhibition by CNS myelin, and it was also shown that CPB/p300 expression is suppressed in the presence of myelin (Gaub et al., 2010). Lastly it was shown that CPB/p300, together with p300-CBP-associated factor (P/CAF), induces acetylation of p53 (Gaub et al., 2010), which has been shown to form a signaling complex with CPB and P/CAF and promote transcription of genes with known roles in promoting axonal growth (Di Giovanni et al., 2006; Tedeschi et al., 2009). The data presented in these studies indicated that CBP and CREB-mediated transcription play significant roles in overcoming inhibition by CNS myelin, and these findings supported those reported in an earlier study by Gao et al. (2004) which showed that induction of CREB-mediated transcription is required for cAMP to promote neurite outgrowth in the presence of MAG, and that expression of constitutively active CREB in DRG neurons is sufficient to enhance regeneration of transected dorsal column axons. Having shown that the effects of cAMP were transcription-dependent (Cai et al., 2002; Gao et al., 2004), the work of the Filbin lab logically progressed to the identification of the genes that were responsible for mediating axonal regeneration. In this review, we will highlight the four genes that have been studied to date: arginase I (ArgI), interleukin (IL)-6, secretory leukocyte protease inhibitor (SLPI), and metallothionein (MT), and discuss the collective role of these and other genes in enhancing axonal regeneration.

## Arginase I

The first cAMP-regulated gene product to be investigated in the context of MAG and myelin-mediated inhibition of neurite outgrowth was ArgI, the rate-limiting enzyme of polyamine synthesis (Cai et al., 2002). ArgI catalyzes the hydrolysis of arginine to ornithine, which is converted to putrescine by ornithine decarboxylase, and putrescine in turn is hydrolyzed to spermidine and spermine (Munder, 2009). In cerebellar granule neurons (CGN) treated with dbcAMP, levels of ArgI mRNA and protein were visibly increased and this was accompanied by a corresponding increase in putrescine synthesis. To assess the importance of increased ArgI expression in enhancing neurite outgrowth, ArgI was overexpressed in CGN using adenoviruses and this allowed these neurons to extend neurites on monolayers of MAG-expressing Chinese hamster ovary (CHO) cells, which indicated that ArgI activity was sufficient to mediate this response. Similarly, the role of polyamine synthesis was investigated by administering two compounds that block the synthesis of these molecules: N (omega)-hydroxynor-L-arginine 5 (NOHA), an inhibitor of ArgI (Boucher et al., 1994), and DL-2-difluor-omethyl-ornithine (DFMO), which inhibits ornithine decarboxylase (Slotkin et al., 1982). Treatment with either NOHA or DFMO abolished the ability of dbcAMP to overcome inhibition by MAG and myelin, and this in turn could be reversed by the addition of putrescine to the cultured neurons, which suggested that polyamines were directly responsible for promoting neurite outgrowth in the presence of myelin-associated inhibitors. The first evidence to support this was provided by experiments showing that putrescine can overcome MAG inhibition either through direct addition or through priming, a procedure in which the neurons were treated overnight with putrescine prior to transferring them to CHO cell monolayers (Cai et al., 2002). It was subsequently shown that putrescine must be converted to spermidine to have this effect, as treatment with the spermidine synthase inhibitor bis-cyclohexylammonium sulfate (BCHS) blocked the ability of putrescine to enhance neurite outgrowth (Deng et al., 2009). Priming neurons with spermidine led to dose-dependent increases in neurite outgrowth in the presence of either MAG or myelin, and more importantly, regeneration of retinal ganglion cell axons in the injured optic nerve was significantly increased following a single intravitreal injection of 20 μM spermidine (Deng et al., 2009). This conclusively demonstrated that the products of ArgI activity can directly promote axonal regeneration *in vivo*.

## Interleukin-6

Expression of the pleiotrophic cytokine IL-6 was found to be strongly induced by dbcAMP in DRG neurons in a microarray performed in collaboration with Jason Carmel, then a member of the laboratory of Dr. Ronald Hart (Cao et al., 2006). IL-6 is a member of the gp130 cytokine family, and other members of this family such as leukemia inhibitory factor and ciliary neurotrophic factor have been shown to have roles in promoting axonal regeneration (Cafferty et al., 2001; Müller et al., 2007). Delivery of IL-6 was found to promote both neurite outgrowth in the presence of myelin-associated inhibitors and *in vivo* axonal regeneration in the injured spinal cord (Cafferty et al., 2004; Cao et al., 2006; Leibinger et al., 2013). Retinal ganglion cells (RGCs) can overcome inhibition by myelin following IL-6 treatment and IL-6 has the added benefit of being neuroprotective, which allowed intravitreal injection of IL-6 to promote axonal regeneration after optic nerve crush (Leibinger et al., 2013). Though this pro-regenerative function is an unexpected finding for a pro-inflammatory cytokine, it brings up the intriguing point that restricted inflammation maybe a positive factor in promoting axonal growth. Though little has been elucidated about IL-6’s mechanism of action, intravitreally injected IL-6 was shown to elevate phosphorylation of signal transducer and activator of transcription (STAT)-3 in the ganglion cell layer (Leibinger et al., 2013). STAT-3 activation triggers neurite outgrowth in both PC-12 and Neuro2A cells (Ihara et al., 1997; Zorina et al., 2010) and overexpression of STAT-3 in RGCs promotes regeneration when an optic nerve crush is performed in combination with lens injury (Leibinger et al., 2013). The latter suggests that STAT-3 alone is not sufficient to promote axonal regeneration and that additional stimuli are required. Hence, further elucidation of the mechanisms and downstream pathways involved with IL-6 dependent axonal regeneration could lead us to potential targets that could promote regeneration without exacerbating the hyper-inflammatory response that is elicited after injury to the CNS.

## Secretory Leukocyte Protease Inhibitor

The same microarray that identified IL-6 also showed that expression of SLPI was increased 3.9-fold in response to elevation of cAMP, and subsequent quantitative real-time PCR analysis of these samples revealed an 8.5-fold increase in SLPI mRNA levels (Hannila et al., 2013). These results were later confirmed in experiments that showed that SLPI mRNA levels were significantly increased following either exposure to dbcAMP or a peripheral conditioning lesion (Hannila et al., 2013). While SLPI was one of the most highly expressed genes identified in the microarray, its role promoting in neurite outgrowth was not immediately apparent, as SLPI is serine protease inhibitor best known for its anti-microbial, and anti-inflammatory functions. These include inhibiting the growth of bacteria, blocking HIV infection of monocytes/macrophages, and inhibiting the expression of the pro-inflammatory cytokines such as tumor necrosis factor α. In the CNS, elevated expression of SLPI has been observed in response to cerebral ischemia, and in a study by Wang et al. (2003), infarct volume was significantly reduced when SLPI was adenovirally overexpressed prior to middle cerebral artery occlusion. SLPI expression was also strongly upregulated following spinal cord contusion injury in mice, and administration of recombinant SLPI produced significant improvement in locomotor function, as well as increased tissue preservation and axonal density, in these animals (Ghasemlou et al., 2010). The findings of these studies have led to the hypothesis that SLPI is neuroprotective, and this has now been complemented by our work showing that SLPI has pro-regenerative effects as well.

In neurite outgrowth assays, neonatal DRG and cortical neurons treated with SLPI were able to overcome inhibition by MAG and myelin, and adult DRG neurons that received intrathecal delivery of SLPI showed enhanced neurite outgrowth in the presence of MAG compared to neurons that received infusions of sterile saline (Hannila et al., 2013). To provide definitive proof that SLPI could overcome inhibition by CNS myelin *in vivo*, a single intravitreal injection of SLPI (10 μg) was administered to adult rats immediately after optic nerve crush. When axonal regeneration was assessed 2 weeks later, there was a significant increase in axonal density distal to the lesion site in animals that received SLPI, which indicated that SLPI can promote axonal regeneration in the injured mammalian CNS.

The importance of SLPI in axonal regeneration is also demonstrated by its role in the conditioning lesion effect. When compared to wild type mice, SLPI null mutant mice displayed significantly less regeneration of dorsal column axons in response to a sciatic nerve lesion (Hannila et al., 2013). This indicated that SLPI is required for this response, and the underlying mechanism can be tied to the expression of Smad2, an intermediate in the transforming growth factor β signaling pathway that is essential for mediating inhibition by CNS myelin. Smad2 can be effectively knocked down using siRNA, leading to increased neurite outgrowth on myelin, and both dbcAMP and sciatic nerve lesions have similar effects, producing significant reductions in Smad2 levels within 18–24 h. In SLPI null mutant mice that received a sciatic nerve lesion, Smad2 expression was unaffected, which indicated that the ability of cAMP to reduce Smad2 is SLPI-dependent. It was also shown that SLPI is capable of rapidly localizing the nuclei of neurons and binding to the promoter for Smad2 with a high degree of specificity, which led us to conclude that cAMP induces expression of SLPI and that SLPI then facilitates axonal growth by blocking transcription of the Smad2 gene and reducing the amount of Smad2 available to mediate inhibition. It is therefore likely that both SLPI and Smad2 could prove to be viable targets for therapeutic intervention in spinal cord injury.

## Metallothionein

MT were first identified nearly 60 years ago, are found in all cells of the body, and though they have been extensively studied, have a poorly defined physiological role. They are distinguished by their small size (approximately 6–7 kD) and abundance of cysteines (20 residues) which coordinate binding of up to seven divalent cationic metals, such as zinc, copper or cadmium (Chung et al., 2008). It is this metal binding ability that has often led to them being labeled as simple chelators of excessive non-protein bound zinc or copper. However, several reports have supported a more selective role for MT in that they could modulate the activity of zinc-dependent proteins, such as transcription factors, by either donating or removing the zinc moiety (Zeng et al., 1991). There are four isoforms of MT: MT-I, -II, -III and -IV; and of these isoforms only MT-IV is not found in the CNS. MT-I and -II are two very closely related isoforms and they are often co-expressed. It is the astrocytes that predominantly express MTs and have been shown to secrete MTs in response to injury (West et al., 2008; Miyazaki et al., 2011). Neurons also express MTs, but at levels that are 2–3 fold lower than glial cells. Extensive studies have been conducted in the nervous system of MT-deficient mice, and these have shown that MT-deficient mice display extremely poor outcomes in models of stroke, seizures, and multiple sclerosis (Carrasco et al., 2000; Penkowa et al., 2001; Trendelenburg et al., 2002). It was through these studies that a neuro-protective role for MTs in the CNS was suggested, and it is believed that MT mediates this effect by scavenging reactive oxygen species (Penkowa et al., 2000; Kondoh et al., 2001).

In our microarray analysis, we observed a significant increase in expression of MT-I/II following either dbcAMP treatment in neonatal DRG neurons or a conditioning lesion in adult DRGs (Siddiq et al., 2015). Though MT-I/II levels have been shown to be elevated in response to divalent metals and glucocorticoids, it was intriguing to see that it was induced in response to elevated levels of cAMP. Though the promoter of MT-I/II lacks a clear cAMP response element, there are several IL-6 response elements. It is therefore possible that the cAMP-dependent increase in IL-6 levels subsequently leads to elevated expression of MT-I/II, and this is supported by the fact that IL-6-deficient mice display reduced expression of MT-I/II after injury to the CNS (Penkowa et al., 2000).

In our study, MT-I/II applied to primary neurons can overcome MAG- or myelin-mediated inhibition and MT-I/II-deficient mice have an attenuated response to conditioning lesion compared to wild-type littermates (Siddiq et al., 2015). Intravitreal injection of MT-I/II also promotes modest axonal regeneration following optic nerve crush, and this pro-regenerative effect is amplified with lens injury (Siddiq et al., 2015), which implies that a combination of MT-I/II and other regeneration-associated genes are required for robust axonal regeneration. Mechanistically, zinc chelation had no effect on myelin-mediated inhibition of neurite outgrowth, suggesting that this was not the source of MT-I/II’s effects (Siddiq et al., 2015). However, MT-I/II directly inhibited the activity of α-secretase, and it also blocked MAG-induced phosphorylation of protein kinase C and activation of the small GTPase RhoA (Siddiq et al., 2015), which suggests that MT-I/II enhances axonal regeneration by interfering with the downstream signaling pathways activated by myelin-associated inhibitors.

## Summary

**Figure 1:**
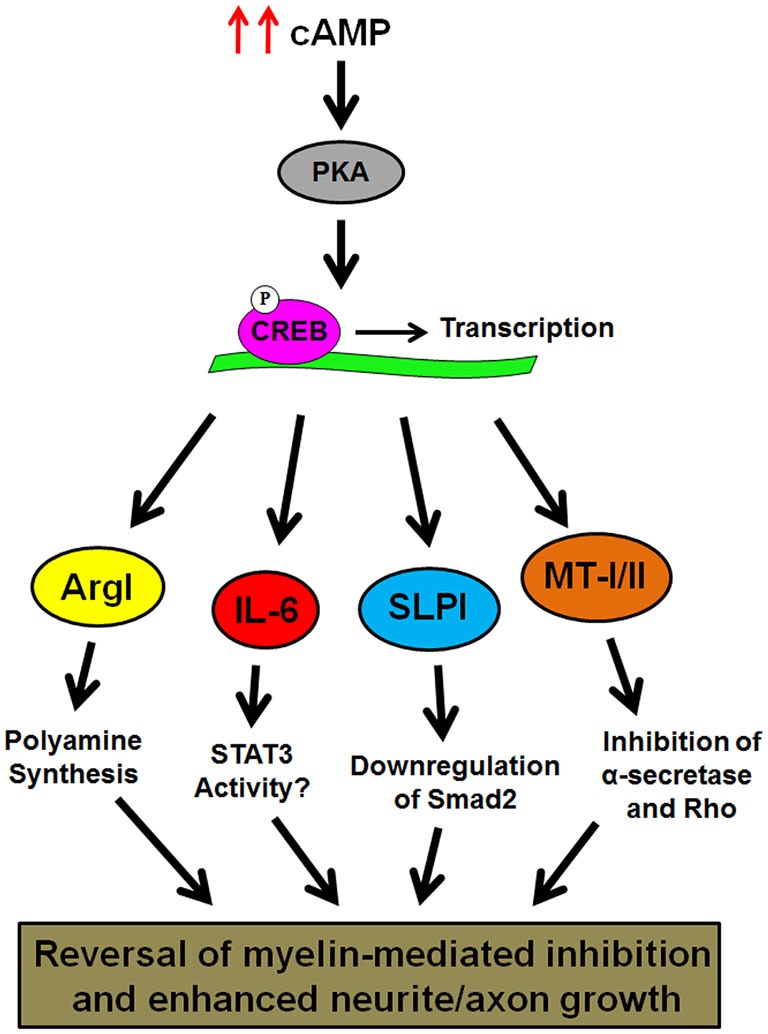
**Schematic representation of cAMP-induced gene expression that has been shown to overcome inhibition by CNS myelin**. Elevation of intracellular cAMP leads to activation of protein kinase A (PKA), and transcription of the ArgI, IL-6, SLPI, and metallothioneins (MT)-I/II genes by phosphorylated CREB. The products of these genes then reverse myelin-induced inhibition of neurite outgrowth through their own distinct mechanisms.

It has become increasingly clear that there is no single pathway, protein, or drug that can promote robust axonal regeneration in the injured CNS, but our microarray analysis of the conditioning lesion effect has provided us with valuable insight into the spectrum of genes that are modulated to produce axonal regeneration. In the case of ArgI, IL-6, SLPI, and MT-I/II, each protein can overcome inhibition by myelin and promote modest axonal regeneration, but all four have uniquely different mechanisms for overcoming the inhibitory environment of the CNS (Figure 1). Another gene that is likely contributing to the conditioning lesion effect is brain-derived neurotrophic factor (BDNF). Like the genes identified in the microarray, BDNF expression is regulated by cAMP and CREB (Finkbeiner et al., 1997), and BDNF has been shown to significantly enhance survival of axotomized neurons following spinal cord injury, and promote axonal sprouting and regeneration (Kobayashi et al., 1997; Kwon et al., 2002). To develop effective treatments for spinal cord injury, it will be necessary to examine the combinatorial effects of IL-6, ArgI, SLPI and/or MT-I/II, as well as other growth-promoting agents such as BDNF, and optimize the dosages to achieve synergistic effects.

In the microarray, we also identified other genes that are upregulated in response to elevated cAMP, such as fibulin 5, superoxide dismutase 3, nerve growth factor inducible protein (VGF), and lactate dehydrogenase A (full microarray results available at http://genome.rutgers.edu/slpi/, Hannila et al., 2013), and their roles in axonal growth have not yet been explored. It should also be noted that numerous genes were significantly down-regulated, such as insulin-like growth factor-binding protein 5, monocyte chemoattractant protein-1, and intriguingly, MT-III (Hannila et al., 2013),^1^ which has been suggested to be inhibitory to neurite outgrowth (Uchida et al., 1991). This suggests that cAMP may not only upregulate the expression of growth-promoting genes, but also limit the expression of genes that negatively impact axonal regeneration. By continuing to investigate the effects of these cAMP-regulated genes on axonal growth, we will advance our understanding of endogenous repair mechanisms, move closer to the ultimate goal of developing methods to enhance them and produce clinical benefit for patients with spinal cord injury.

## Author Contributions

MS and SH conceived, wrote, and critically revised the manuscript.

## Conflict of Interest Statement

Mustafa M. Siddiq declares that the research was conducted in the absence of any commercial or financial relationships that could be construed as a potential conflict of interest. Sari S. Hannila holds United States Patent 8,367,615, “Stimulation of neuron regeneration by secretory leukocyte protease inhibitor”.

## Footnotes

^1^http://genome.rutgers.edu/slpi/
